# Micro-Fabricated DC Comparison Calorimeter for RF Power Measurement

**DOI:** 10.3390/s141120245

**Published:** 2014-10-27

**Authors:** Bilel Neji, Jing Xu, Albert H. Titus, Joel Meltzer

**Affiliations:** 1 Department of Electrical Engineering, University at Buffalo, The State University of New York, Buffalo, NY 14260, USA; E-Mails: bilelnej@buffalo.edu (B.N.); jingxu@buffalo.edu (J.X.); 2 Bird Technologies, Solon, OH 44139, USA; E-Mail: JMeltzer@bird-technologies.com

**Keywords:** RF power sensor, micro-calorimeter, microfluidics, temperature sensor, DC comparison

## Abstract

Diode detection and bolometric detection have been widely used to measure radio frequency (RF) power. However, flow calorimeters, in particular micro-fabricated flow calorimeters, have been mostly unexplored as power meters. This paper presents the design, micro-fabrication and characterization of a flow calorimeter. This novel device is capable of measuring power from 100 *μ*W to 200 mW. It has a 50-Ohm load that is heated by the RF source, and the heat is transferred to fluid in a microchannel. The temperature change in the fluid is measured by a thermistor that is connected in one leg of a Wheatstone bridge. The output voltage change of the bridge corresponds to the RF power applied to the load. The microfabricated device measures 25.4 mm × 50.8 mm, excluding the power supplies, microcontroller and fluid pump. Experiments demonstrate that the micro-fabricated sensor has a sensitivity up to 22 × 10^−3^ V/W. The typical resolution of this micro-calorimeter is on the order of 50 *μ*W, and the best resolution is around 10 *μ*W. The effective efficiency is 99.9% from 0–1 GHz and more than 97.5% at frequencies up to 4 GHz. The measured reflection coefficient of the 50-Ohm load and coplanar wave guide is less than −25 dB from 0–2 GHz and less than −16 dB at 2–4 GHz.

## 1. Introduction

Radio frequency (RF) power measurement is very important in many applications, such as lab equipment calibration, communication systems, automotive control systems, satellites and robots. Accurate RF power measurement continues to be challenging and is essential to ensure consistency across different devices under all conditions, and it therefore plays a critical role in the performance, repeatability and traceability of RF devices. Up to now, RF power has been mainly measured using diode or bolometric detectors, such as thermistors and thermocouples [1,2].

**Figure 1.**
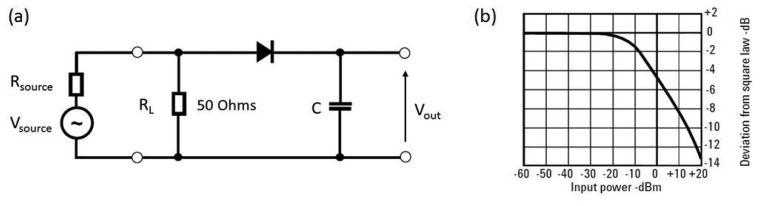
(**a**) Diode detector circuit; (**b**) diode detector characteristics showing square-law and linear region.

Diode detectors use the rectification property of diodes to measure RF power. The obtained DC voltage is simply proportional to the RF power level of the signal. Figure 1a represents the circuit of a simple diode detector [1]. Typically, diodes used for rectification are low-barrier Schottky diodes, due to their superior performance at high frequencies. These diodes can only be used for power measurement in the square law region, where the rectified output is proportional to the square of the input signal voltage. It can be seen from Figure 1b that the square-law detection region exists from the noise level (typically −70 dBm) up to approximately −20 dBm [1]. As the power level increases beyond −20 dBm, the diode moves into the linear region, where the rectified output voltage is proportional to the input voltage and, hence, not well suited to power measurement.

The frequency range of diode RF power sensors is from 10 MHz up to 50 GHz. Diode detectors are the most widely used commercial RF power sensors [1,3].

The second most commonly used RF power sensors are thermistors. A thermistor is a resistor whose electrical resistance changes with temperature [2,4] and is usually made of a metallic oxide compound. The resistance of a negative temperature coefficient (NTC) device decreases with temperature rise, and the resistance of a positive temperature coefficient (PTC) device increases with temperature rise [5].

Thermocouples have been extensively used for power measurement. When two different metals are connected together to form a junction and the junction is heated, the potential across the free ends is proportional to the temperature difference between the hot and cold junctions. This phenomenon is called the Seebeck effect [5].

**Figure 2.**
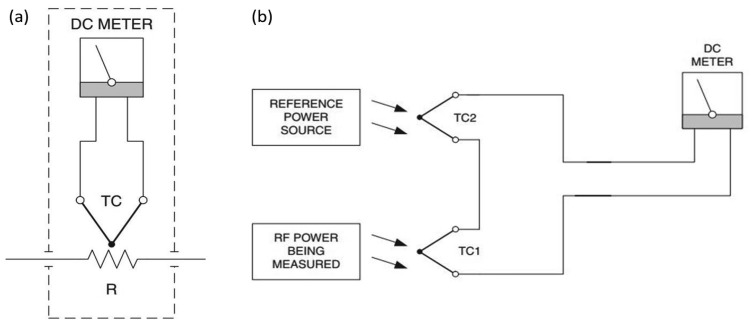
(**a**) Basic thermocouple power sensor; (**b**) thermocouple power sensor with a compensating thermistor.

As shown in Figure 2a, a thermocouple is placed adjacent to the load inside a thermally-sealed housing. the potential across the ends of the thermocouple changes proportional to the incident RF power. A compensating junction is however used as a reference to account for ambient variations in temperature, as presented in Figure 2b. Thermocouples have been used for higher frequency power meters since the 1970s. They are considered more sensitive than thermistor sensors and are inherently square law devices [2,4].

In addition to the above described power measurement methods, a few MEMS-based RF power sensors have been introduced in [6,7].

There are a number of RF power calorimeters on the market today. For example, Electro-Impulse, Inc. (Neptune, NJ, USA), produces a line of meters that are based on calorimetric measurements; these higher power meters (15 W to 80 kW) weigh between 70 and 600 pounds, depending on the model [8]. Another example is the Bird-6090 Calorimeter that was introduced in 1983 by Bird Technologies. It operates at a frequency range between DC and 3.5 GHz, and its dimensions are 293.7 × 148 x 451 mm^3^ and the weight 36 lbs. Additionally, the meter stabilization time is three minutes [9].

Although these high quality devices are available, there is a need for smaller and more portable devices for field use that are traceable to NIST standards. Because the calorimetric method of measurement can significantly reduce the errors in the transfer standard, the calorimeter has long been considered the most accurate method for high-level RF power measurement. However, this technique has a few limitations, such as the amount of time needed to reach equilibrium and the difficulty in measuring low power. These limitations can be minimized or eliminated by the use of micro-fabrication to make a calorimeter. The resulting microfabricated device can have a well-matched load and a fast response time, because it has very low thermal mass. While such micro-fabricated calorimeters have been extensively used for determination of thermo chemical values and reaction kinetic studies [10], micro-fabricated calorimeters as power sensors have yet to be fully developed or explored.

In this paper, the miniaturization of a flow calorimeter is demonstrated. Experimental results, such as RF power measurement, the impact of using fluid on heat transference and DC comparison measurement, are presented. The experimental results demonstrate the ability to measure RF power from 100 μW to 200 mW over frequencies ranging from DC to 5 GHz. The microcalorimeter measures 25.4 mm x 50.8 mm, weighs less than one pound and has a response time of less than one minute. The resolution of the self-heating power sensor described in [11] is on the order of 0.01 mW, which is comparable to the best resolution of our micro-calorimeter. On the other hand, the average sensitivity of our micro-calorimeter is 22 × 10^−3^ V/W. This sensitivity is higher than the 3.4 × 10^−3^ V/W in [11] and the 0.16 × 10^−3^ V/W in [12] of the proposed self-heating power sensors. Our sensitivity is also higher than the 0.85 × 10^−3^ V/W reported in [13] and the 0.2 × 10^−3^ V/W in [14] of the reported GaAs-based indirect-heating power sensors.

## 2. Micro-Calorimeter Design and Fabrication

### 2.1. Flow Calorimeter Principle

**Figure 3.**
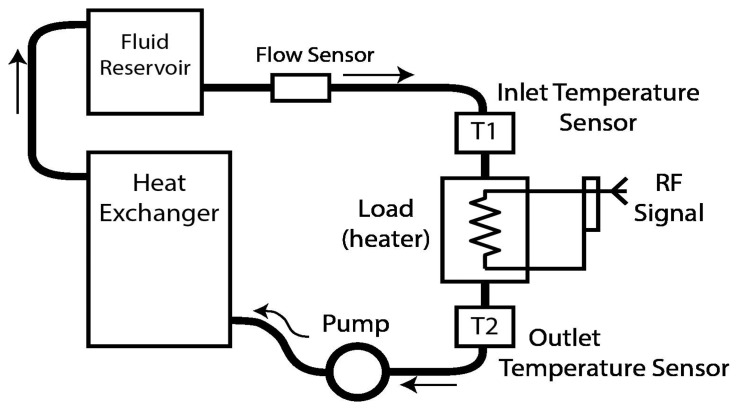
RF power calorimeter block diagram.

The principle of the RF power calorimeter is based on the first law of thermodynamics, which states that energy cannot be created nor destroyed, only converted from one form to the other. A calorimeter uses this concept to transduce RF power into thermal energy for measuring RF power. The RF power to be measured is passed to a resistive load, causing a temperature rise in the load and its surroundings. A fluid in contact with the load carries the heat to thermistors where a temperature rise is measured [15,16]. A system level block diagram of a typical absolute-flow RF calorimeter is shown in Figure 3 [17].

RF power is passed to the internal load resistor (labeled ‘Load’ in Figure 3). The load resistor is hollow and has the working fluid flowing through it. A pump is used to circulate the fluid from the reservoir to the load and then back to the reservoir after removing the heat by passing it through a heat exchanger. The reservoir is large enough to stabilize the working fluid and maintain an average constant temperature. Temperature sensors are used to measure the temperature difference between the fluid before passing through the load and after. The sensors are mostly thermistors, due to their fast response and sensitivity, and a flow sensor is used to determine the mass flow rate of the fluid through the load. By using thermodynamic first principles, the RF power can be determined from the temperature difference, mass flow rate and specific heat of the fluid.

### 2.2. Proposed Flow Calorimeter Design

**Figure 4.**
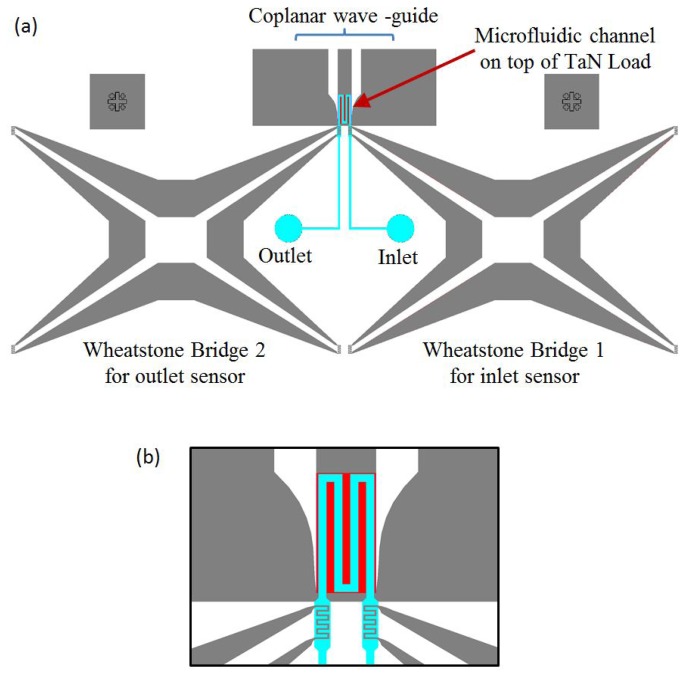
(**a**) RF power micro-calorimeter design; (**b**) zoomed view showing the structure of the load (red rectangle), sensors (serpentine structure underneath microfluidics) and microfluidic channels (cyan).

The physical design of the micro-fabricated calorimeter is shown in Figure 4a. It consists of a tantalum nitride thin film load, a coplanar wave-guide and two temperature sensors made from aluminum (Al), all of which are deposited on a glass substrate. A microfluidic channel with a serpentine structure bonded on the load is used to transfer heat; the heat is generated by the applied power to be measured [18]. An inlet sensor is included to measure the initial temperature of the fluid, and the output sensor measures the final temperature after heating.

Details of the load, temperature sensors and the serpentine structure of the microfluidic channel are shown in Figure 4b. Glass is used as a substrate to carry the load and sensors. It is chosen due to its very low thermal conductivity, which is 100-times smaller than that of silicon. Lower thermal conductivity is required to limit unwanted heat loss through the substrate to the surroundings. Glass also has a much better electromagnetic response than silicon, as the high frequency performance of the load depends very much on the dielectric strength of the substrate. The ideal load is required to be a thin film resistor with uniform material properties, low temperature coefficient of resistance (TCR), long-term stability and low nonlinear effects. For good RF performance with lower reflected power, the resistance needs to be accurate and uniform. Tantalum nitride (TaN) is chosen as the material for the load, due to its low TCR. For ease of prototype fabrication, a coplanar wave-guide (CPWG) structure is constructed, using TaN for the load and Al for the ground plane, to direct the RF power to the load.

**Figure 5.**
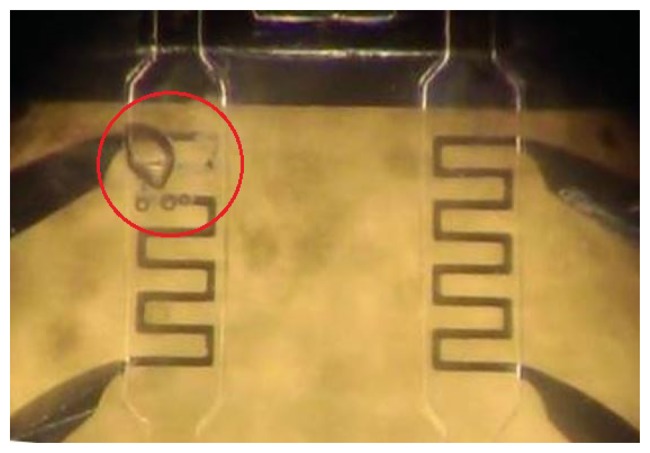
The effect of deionized water on the temperature sensor's patterns.

Aluminum-resistive temperature detectors (RTDs) are used as temperature sensors. RTDs are very suitable for a microfluidic design, as they can be made to have much better contact with the fluid, due to their simple planar structure. Aluminum is chosen due to its low cost, its linearity and relatively high TCR [19]. Since it is necessary to measure small changes in resistance, Wheatstone bridges are used [20]. An MP6 micro-pump is used to pump the fluid. Although deionized water could be used as the fluid, it was decided to use HFE-7500. Compared to deionized water, HFE-7500 has a higher viscosity and less reaction with aluminum, which results in less corrosion and fewer pattern defects. From the experiment results, we see that when deionized water is used, after a few minutes of operation, the metal of the temperature sensor begins to disappear and its electrical resistance increases dramatically (see Figure 5). On the other hand, when HFE-7500 is used, we see no corrosion when in contact with the sensor, and the patterns remain undamaged, allowing the devise to operate for many days without a problem.

### 2.3. Related Mathematical Model

**Figure 6.**
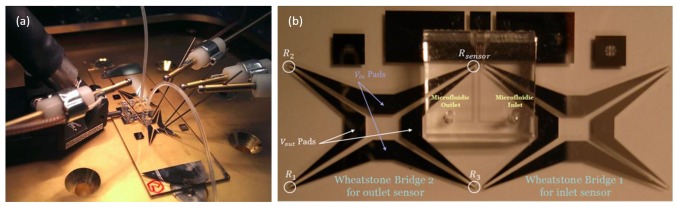
(**a**) Micro-fabricated calorimeter connected to DC probes and the RF probe; (**b**) top view of the micro-fabricated sensor.

The Wheatstone bridge is mainly composed of four resistances and four pads. As shown in Figure 6b, *R*_1_, *R*_2_ and *R*_3_ are not in contact with the fluid. They should be matching all the time, and they are not supposed to be affected by the temperature change in the fluid. On the other hand, *R_sensor_* is directly related to the fluid temperature change. One pair of the pads on the bridge is used to apply a DC voltage (*V_in_* = 0.5V) and the other pair is used to measure the output voltage. The typical temperature dependent first order function for the resistance of metals is represented by the following equation [21]:

(1) $$R_{\text{sensor}}(T_{\text{sensor}})=R_{\text{sensor}0}*(1+\text{TCR}0*T_{\text{sensor}})$$

where *R_sensor_* and *R_sensor_*_0_ are the sensor's resistances at temperatures *T_sensor_* and 0 °C, respectively, and *TCR*0 is its temperature coefficient of resistance at 0 °C: TCR0 = 0.0039 K^−1^; this value is derived from the literature. The sensor's temperature can be extracted from (1) as:

(2) $$T_{\text{sensor}}=(\frac{R_{\text{sensor}}(T_{\text{sensor}})}{T_{\text{sensor}0}}-1)*\frac{1}{\text{TCR}0}$$

The relation between *V_out_, V_in_* and *R_sensor_* is represented by the following equation:

(3) $$V_{\text{out}}(T_{\text{sensor}})=(\frac{R_{\text{sensor}}(T_{\text{sensor}})}{R_{3}+R_{\text{sensor}}}-\frac{R_{2}}{R_{1}+R_{2}})*V_{in}$$

An applied power on the load will generate a heat that is transferred via the fluid to the outlet sensor, resulting in an increase in its resistance. Therefore, *V_out_*(*T_sensor_*) increases accordingly.

### 2.4. Micro-Fabrication Process

All micro-fabrication is done using the clean room facilities at the University at Buffalo. To prepare the coplanar wave-guide, interconnection and temperature sensors, a positive photoresist (Shipley 1818) is spin-coated on a glass slide and patterned using a conventional UV photolithography process. Then, aluminum is deposited via E-Beamevaporation. A layer of Shipley 1818 positive photoresist is coated again to cover all patterns and exposed to create the load pattern. DC sputtering is then used to deposit TaN into the photoresist mold, after which acetone is used to enable lift-off.

Polydimethylsiloxane (PDMS) is used for creating the microfluidic channels and connecting the external pump. First, a layer of negative photoresist (MicroChem, SU-8 2050) is spin-coated and patterned on a silicon wafer. After developing the UV exposed photoresist, a soft mold is formed. Then, PDMS mixture is poured over the soft mold, followed by curing at 80 °C for half an hour. Finally, a PDMS replica is punched to form the inlet and outlet, followed by *O*_2_ plasma treatment for 20 s to bond it irreversibly with the glass substrate [22]. The width of the channels is 100 *μ*m.

Figure 6a shows the fabricated device under testing. An RF probe is applied to the co-planar wave guide to apply RF power. DC probes are used to drive the Wheatstone bridge and read its output voltage, which is a function of the applied RF power. The description of the fabricated micro-calorimeter is presented in Figure 6b.

### 2.5. DC Comparison Method

In order to determine the average power of an unknown RF source, the output voltage of the Wheatstone bridge, corresponding to a portion of the heat generated by the RF source, which is transferred from the load to the sensor, is measured and converted to a digital value using an analog-to-digital converter. The measured value is saved in the internal memory of the micro-controller. The output voltage corresponding to the unknown applied power is called *V_out_* – *RF*. Then, a small known DC power is applied to the load, and the corresponding output voltage from the bridge is measured (called *V_out_* – *DC*), saved in the internal memory of the micro controller and compared to *V_out_* – *RF*. When *V_out_* – *DC* is smaller that *Vout* – *RF*, then the known DC power is increased until *V_out_* – *DC* is equal to *V_out_* – *RF*, which means that the output temperature corresponding to the unknown RF power is substantially equal to the output temperature corresponding to the known DC power [23,24]. In this case, the unknown applied RF power is equal to the known substituted DC power.

**Figure 7.**
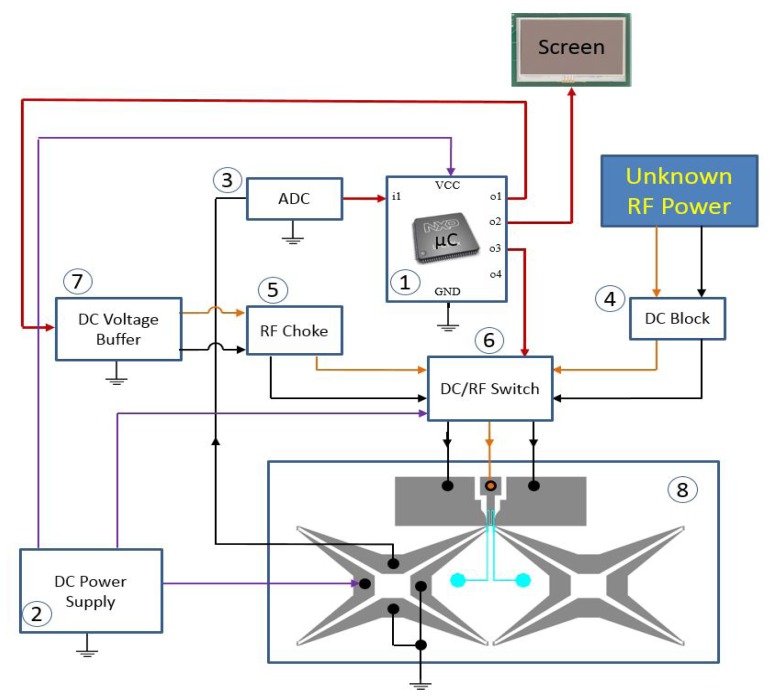
DC comparison diagram: red wires correspond to digital signals, and orange wires correspond to applied RF and DC power to the load.

Figure 7 shows the block diagram of our entire DC comparison calorimeter system whose purpose is to measure an unknown applied power using a comparison to a known DC voltage level. The measurement system is composed of:

(1) The LPC1768 microcontroller (*μ*C) based on the ARMCortex-M3 with low power dissipation. This is used to control the RF/DC switch, to adjust the DC power level and to display the output on an LCD screen.
(2) The Tektronix PS2521 power supply. This is used to generate a 0.5 V supply that is the input to the Wheatstone bridge, a 12-V supply to the RF/DC switch and a 3.3-V supply for the *μ*C
(3) A 24-bit TI ADS1232 analog-to-digital converter. This is used to convert the analog output voltage of the Wheatstone bridge to a digital value for the *μ*C
(4) DC block. This is used to prevent any DC power from going to the RF source.
(5) RF choke. This is used to block any RF power from affecting the DC source.
(6) DC/RF switch. This is used to direct the unknown RF power or the known DC power to the load.
(7) DC voltage buffer.
(8) Microfabricated calorimeter with Wheatstone bridges.

National Instruments' LabView is used to record data, such as the applied DC power to the bridge and the load and the output voltage of the Wheatstone bridge during the testing of the system. As described, the LCD screen is used to show the different DC comparison measurement steps and displays the final value of the unknown applied RF power when the measurement is complete.

The *μ*C is programmed to control the operations of the system and record and save various readings. First, the *μ*C sets the DC/RF switch so that the RF power is applied to the load. The bridge output is an analog voltage that corresponds to an unknown RF power applied to the load and is converted to a digital value by the ADC before being sent to the *μ*C This value is saved by the *μ*C for later comparison. After the reading is made, the *μ*C signals the DC/RF switch to connect the known DC power to the load. This known voltage now generates heat, which is then transferred to the fluid and then changes the temperature of the thermistor, exactly as the unknown RF power did. This new output voltage from the bridge is digitized and compared to the saved value corresponding to the applied RF power. If the output from the bridge for the known DC power does not match the saved RF power output, then the program running on the *μ*C adjusts the known DC power, which is reapplied to the load, and the corresponding bridge output is read again. This loop of adjusting the known DC power, reading the output and comparing it to the RF power output continues, until the two values match. When the two values match, meaning they are equal to within some tolerance determined by the minimum power step generated by the DAC, then the value of the known DC power that produced the match corresponds to the power of the unknown applied RF signal. This power is then displayed on the LCD screen.

## 3. Experimental Results

In this section, we present the experimental results obtained from testing the individual components of the device and discuss the operation of the system.

### 3.1. Microfabricated Load Impedance Measurements

**Figure 8.**
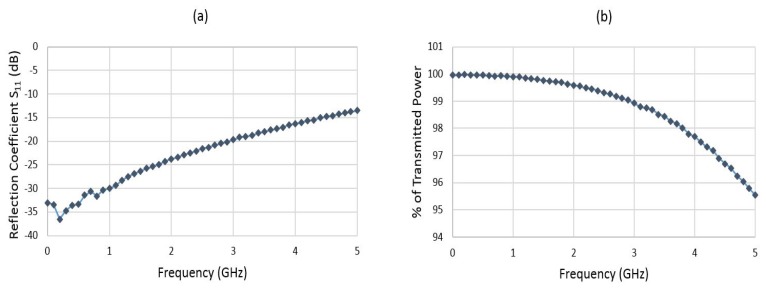
(**a**) Measured reflection coefficient of the fabricated RF power sensor; (**b**) percentage of transmitted RF power *vs.* frequency.

The load and coplanar wave guide impedance has been optimized to minimize reflection and attenuation in order to obtain an impedance of 50 ohm. Figure 8a shows the measured reflection coefficient (S11) of the RF power sensor using the ANRITSU 37397C vector network analyzer. A calibration substrate (GGB CS-18) has been used for accurate RF measurement. The measured S11 is lower than −25 dB at 0-2 GHz and less than −16 dB at 2–4 GHz.

The percentage of transmitted RF power to the load as a function of frequency is presented in Figure 8b. More than 99.9% of the RF power is transmitted at 0– 1 GHz; more than 99.6% is transmitted at 1–2 GHz, and more than 97.5% is transmitted at 2–4 GHz.

### 3.2. Micro-Fabricated Calorimeter Performance

**Figure 9.**
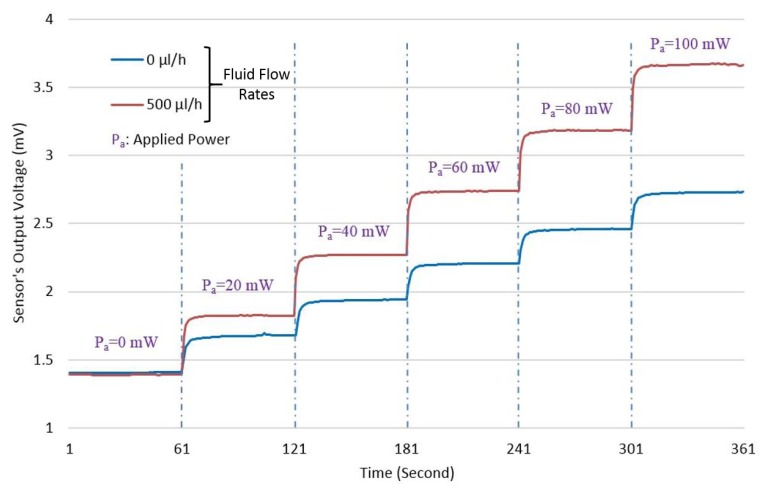
Measured sensor's output voltage *versus* time at different RF power levels from 0 mW to 100 mW. The frequency is 5 GHz. Flow rates are in microliters per hour (*μ*l/*h*).

An initial test of the micro-fabricated device is performed by applying a known RF power to the load and measuring the corresponding sensor's output voltage. Figure 9 shows the device response at 5 GHz, which is the maximum frequency of operation. The total measurement time is six minutes, and the applied power is increased by 20 mW every one minute.

The blue curve in Figure 9 represents the measurement with no fluid flow. Because *V_out_* increases as the applied power increases, which ideally, it should not, it implies that there is a power leakage via the substrate. With an applied flow of 500 *μ*l/h, which is shown by the red curve in Figure 9, we observe a voltage rise compared to the no flow case. Since we are using an inlet sensor as a reference, the voltage rise coming from the substrate can be canceled out when measuring the voltage difference between inlet and outlet. Therefore, only the heat transferred by the liquid contributes to the output voltage.

**Figure 10.**
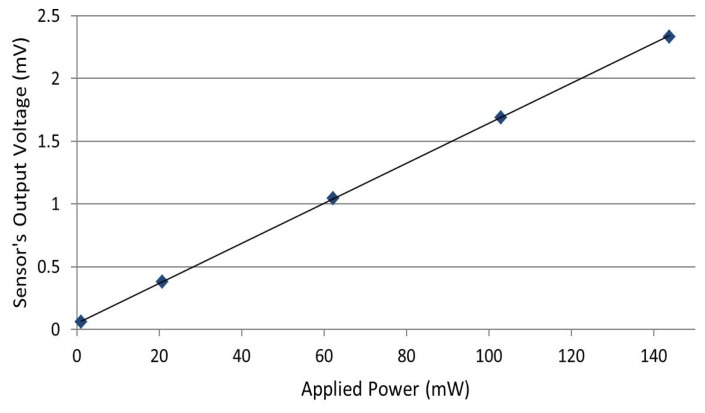
Measured sensor's output voltage versus applied DC power; standard deviation errors are presented in Table 1.

In order to determine the calorimeter linearity, we measure the sensor's output voltage corresponding to different DC powers (20 mW, 60 mW, 100 mW and 140 mW), each applied 20 times, and we calculate the average output corresponding to each power. Figure 10 shows the measured output voltage as a function of applied power. The standard deviation error related to applied power, and the sensor's output voltage is presented in Table 1. Experimental results show a good linearity of the sensor's response with respect to the applied power, with small measurement errors.

It should be stated that the slope of the curve in Figure 10 (*i.e.*, calorimeter sensitivity) is affected by the fluid properties, such as thermal conductivity, viscosity and flow rate. Higher flow rates and thermal conductivity result in more heat transfer from the load to the outlet sensor; therefore, higher output voltage is measured. The sensitivity of the fabricated device is in the range of 3.2 × 10^−3^ V/W to 22 × 10^−3^ V/W. Using water at about 1000 *μ*L/h (a fluid with a high thermal conductivity and high flow rate) gives better sensitivity. On the other hand, using HFE-7500 at 0 *μ*L/h (a fluid with a low thermal conductivity and no flow rate) gives poorer sensitivity. However, from the experiments using water, we noticed that the RTD sensor is damaged, because of aluminum oxidation. Therefore, HFE-7500 is used at the highest flow rate that the micropump can provide to obtain the best sensitivity and not damage the device.

The resolution of the device is determined by applying low DC power, while measuring the sensor's output voltage. A precise voltage source using a micro-controller and a 10-bit digital-to-analog converter has been used. Initially, the voltage source has an offset of 14 mV. Therefore, the applied voltage range is from 14 mV to about 3.3 V with a minimum step of 3.22 mV, the equivalent of 3.9 *μ*W to about 217.8 mW with a minimum step of 0.21 *μ*W.

**Figure 11.**
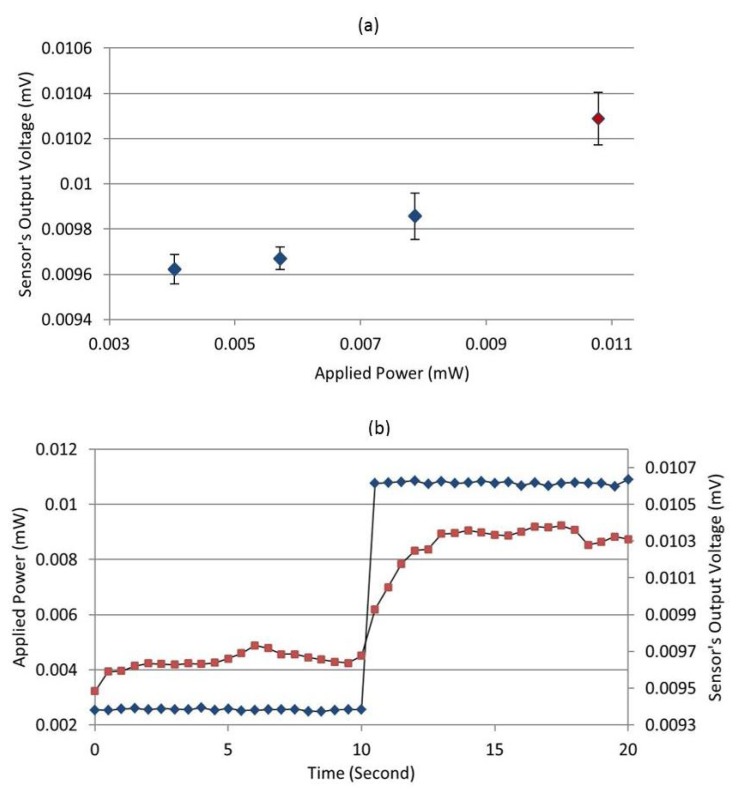
Resolution determination: (**a**) the sensor's output voltage as a function of applied low DC power; (**b**) the sensor's output voltage before applying power and after applying 10.7 mW power.

The typical resolution of the fabricated calorimeter is 50 *μ*W, and the best resolution is about 10 *μ*W. Figure 11a represents a situation where the device has the best resolution. Error bars correspond to the standard deviation of the sensor's output voltage (20 successive measurements for each applied power).

From Figure 11a, we can see that since the error bars of the three first applied powers (blue diamonds) overlap, it is possible that these would be indistinguishable from each other in a real measurement situation. On the other hand, the fourth applied power, which is about 10.7 *μ*W (shown by the red diamond), is significantly different from the first three, so we could distinguish it from lower applied powers. Thus, we conservatively consider this as the minimum resolvable value.

Figure 11b shows the response of the sensor when 10.7 *μ*W is applied to the load. The blue diamonds in Figure 11b show the applied power (left *y*-axis) *versus* time, and the output voltage is shown with the red squares (right *y*-axis). Initially, no power is applied for the first ten seconds, then 10.7 *μW* is applied for the next ten seconds. We can clearly see a change from no power to applied power in the output when the power is applied. From these measurements, we can see that the sensor responds quickly, on the order of a few seconds, and stabilizes after approximately five seconds. This is totally acceptable for RF power generators calibration, where a real-time response is not required.

### 3.3. System Calibration

**Figure 12.**
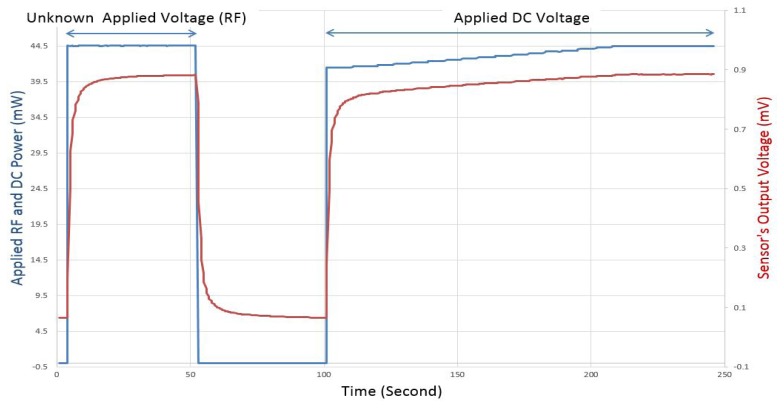
Example of DC comparison: applied RF and DC power and sensor's output voltage as a function of time.

The process for making DC comparison measurements (as shown in Figure 12) is as follows: (i) apply an unknown RF power and read and save the corresponding sensor's output voltage after stabilization (*t* = 0 to 50 s); (ii) apply zero power and wait until the sensor's output voltage goes back to the initial starting point (*t* = 50 to 100 s); (iii) apply a known DC power and keep increasing it until the sensor's output voltage matches the saved voltage value corresponding to the unknown RF power (*t* > 100 s).

For improved accuracy, the stabilization waiting time is variable and changes according to the applied power. For the case represented by Figure 12, the waiting time is set to 50 s for the two first steps of the DC comparison process, and two seconds for each power step increase in the third step of the process.

**Figure 13.**
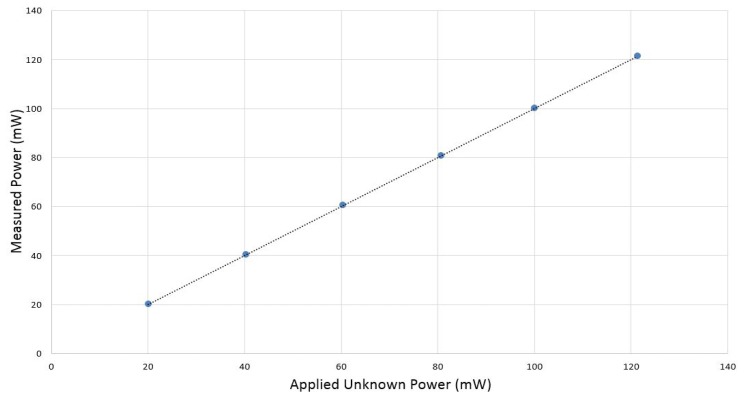
Measured power *versus* unknown applied power.

For each applied power level (20mW, 40 mW, 60 mW, 80 mW, 100 mW and 120 mW), multiple measurements are made, and then, the mean of each measurement is shown as function of applied power in Figure 13.

**Figure 14.**
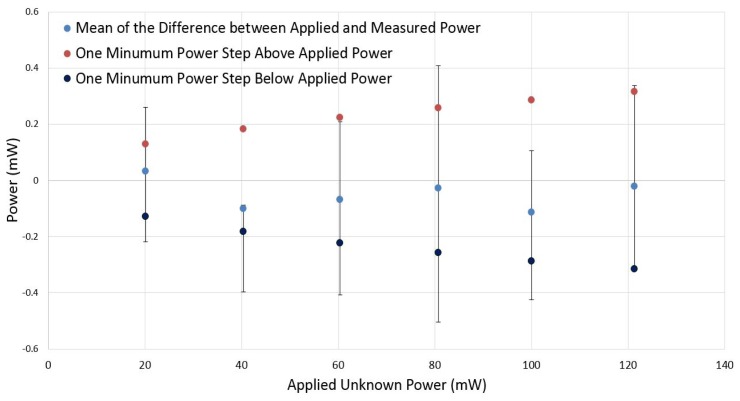
Mean of the difference between applied and measured power *versus* applied unknown power.

The DC power substitution error is determined by the difference between the applied unknown power and measured power. The mean of this error is shown in Figure 14. It is in the range of −0.11 mW to 0.033 mW. Error bars correspond to the minimum and maximum difference between applied and measured power.

The minimum power step above and below applied power is also shown in Figure 14 (red and black data, respectively). These power steps are defined by the resolution of the digital to analog converter. It could be concluded that most of the measurement errors are between two power steps generated by the DAC, which means that most of the DC comparison inaccuracy is related to the limited resolution of the DAC used. It should be noted that the DAC is generating voltage steps with a reasonable resolution (3.22 mV); however, in terms of power (square of voltage), the resolution is getting lower at higher power values.

**Figure 15.**
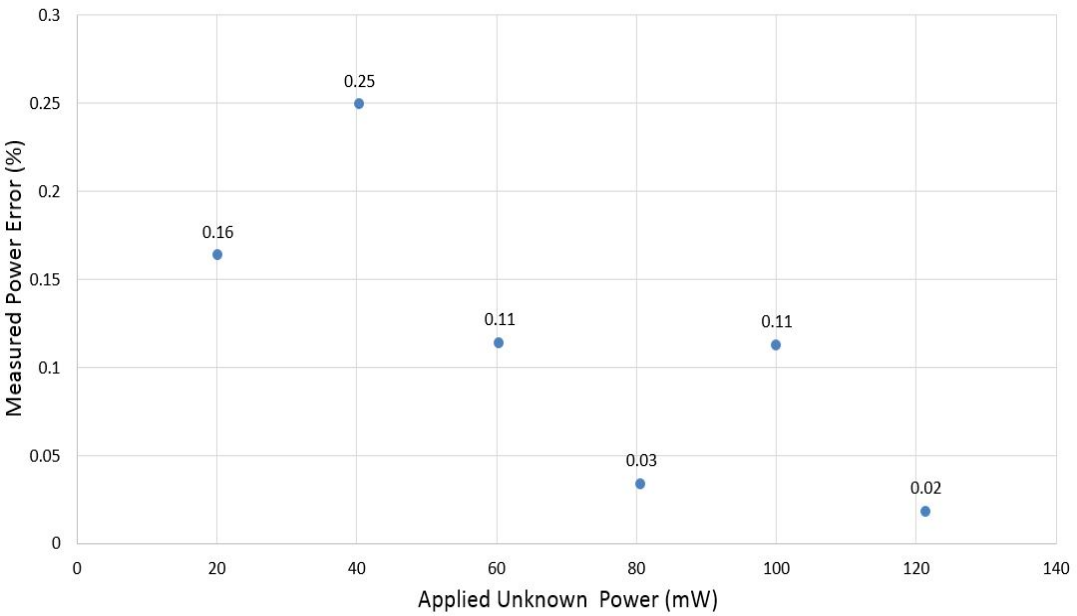
Percentage error of measured power as a function of applied unknown power.

The percentage error of measured power at different applied power is shown in Figure 15. From the obtained results, we see that the accuracy of measurement is higher than 99.7% without considering the error coming from power reflection.

The obtained results demonstrate the importance of using flow calorimeters for RF power measurement. Nevertheless, the performance could be improved by: (i) using a DAC with higher resolution; (ii) using the inlet sensor as a reference and calculate the difference between its output and the outlet sensor's output to eliminate any kind of noise or heat losses; (iii) using a more stable and accurate power source to supply the Wheatstone bridge.

## 4. Conclusions

We demonstrate the operation of a micro-fabricated calorimeter. The device is capable of measuring power from 100 *μ*W to 200 mW at frequencies up to 5 GHz with more than 97.5% accuracy and a sensitivity up to 22 × 10^−3^ V/W. While this was the target power measurement range for our design, the device can measure powers down to the level of the minimum resolution. The upper range on power measurements is limited by the load resistor size, since powers that are too high could damage the thin-film device. The typical resolution of the fabricated sensor is 50 *μ*W and its best resolution is around 10 *μ*W. In addition, the use of DC comparison for power measurement is demonstrated. From the obtained results, The measurement accuracy is higher than 99.7% and could be improved by using a DAC with higher resolution. The next step of this work includes the improvement of load and CPW impedance matching for smaller reflection at higher frequencies.

## Figures and Tables

**Table 1.** Standard deviation error of applied power and the sensor's output voltage.

**Applied Power**		**Sensor's Output Voltage**	

**Average (mW)**	**SD Error (*μ*W)**	**Average (mV)**	**SD Error (*μ*V)**
1.043236	1.261	0.061634	0.872
20.654638	3.359	0.379975	0.677
62.210728	4.674	1.049162	1.181
102.849828	5.553	1.692909	0.717
143.786107	13.523	2.336731	0.841
